# Prognostic value of prognostic nutritional index in patients undergoing surgery for gastric cancer

**DOI:** 10.3389/fsurg.2025.1618111

**Published:** 2025-09-03

**Authors:** Fırat Mülküt, Cem Batuhan Ofluoğlu, Mustafa Kağan Başdoğan, İsa Caner Aydın, Osman Akdoğan, Adnan Gündoğdu, İsmail Ege Subaşı

**Affiliations:** ^1^ General Surgery Department, Martyr Dr. İlhan Varank Sancaktepe Training and Research Hospital, Dudullu¸ Türkiye; ^2^ Gastrointestinal Surgery Department, Zonguldak Atatürk State Hospital, Zonguldak, Türkiye; ^3^ General Surgery Department, Ministry of Health, Keşan State Hospital, Edirne, Türkiye

**Keywords:** gastric cancer, prognostic nutritional index (PNI), signet cell gastric carcinoma, survival, post-operative outcomes

## Abstract

**Background and aim:**

Gastric cancer is among the commonly occurring cancers worldwide and is one of the leading causes of cancer-related deaths. Malnutrition is an important factor affecting the course of disease and treatment response in gastric cancer patients this study aimed to investigate the effect of the Prognostic Nutritional Index (PNI) on postoperative complications and long-term survival in gastric cancer patients, and to comparatively examine PNI values among different histological subtypes.

**Methods:**

Data from patients who underwent curative surgical resection for gastric cancer between 2014 and 2020 were retrospectively analyzed. PNI values were calculated using the formula: 10 × serum albumin (g/dl) + 0.005 × lymphocyte count (cells/mm^3^). The optimal cut-off value for PNI was determined through ROC analysis. The relationship between PNI values and clinicopathological features, postoperative complications, 5-year overall survival (OS), and histological subtypes was evaluated.

**Results:**

A total of 220 patients (161 males, 59 females; mean age: 60.63 ± 10.56) were included in the study. The mean PNI value was 47.15 ± 6.07. ROC analysis established an optimal PNI cut-off value of 46.2 (AUC = 0.673, 95% CI: 0.599-0.747, p<0.001; sensitivity 78.8%, specificity 51.9%). Complication rates were significantly higher in the patient group with PNI < 46.2 (*p* = 0.006). The 5-year OS rate was 30.0%. Patients with low PNI values had significantly shorter survival (log-rank *p* = 0.001). Major complications were more frequent in patients with low PNI (*p* = 0.006). Patients diagnosed with signet ring cell carcinoma (SRCC) had significantly lower PNI values compared to other adenocarcinoma subtypes (*p* = 0.001). PNI values were lower in the presence of perineural invasion (*p* = 0.005) and lymphovascular invasion (*p* = 0.032). In multivariate analysis, tumor stage (for Stage I *p* = 0.01, Stage II *p* = 0.034, Stage III *p* = 0.002) and PNI value (*p* = 0.001) were identified as independent prognostic factors affecting 5-year OS. Conclusion: PNI is an important marker for predicting long-term survival and postoperative complication risk in patients with gastric cancer. The significantly lower PNI values in the SRCC subtype compared to other histological subtypes indicate the necessity of closer monitoring of nutritional status in this patient group. Our results suggest that preoperative PNI assessment could be a valuable parameter in planning patient-specific treatment approaches.

## Introduction

Gastric cancer is among the commonly occurring cancers worldwide and is one of the leading causes of cancer-related deaths (1). Malnutrition is an important factor affecting the course of disease and treatment response in gastric cancer patients (2). Biomarkers such as the Prognostic Nutritional Index (PNI) are objective parameters used to assess the nutritional status of patients. PNI reflects the nutritional status of patients based on serum albumin and total lymphocyte count, and recent studies have demonstrated the prognostic and post-operative value of PNI in gastric cancer patients (3). Additionally, signet ring cell gastric cancer (SRCC) constitutes approximately 8%–30% of all gastric cancers and is a subtype with a more aggressive course and poor prognosis. The tendency of SRCC to occur at a younger age, the risk of late diagnosis, and its resistance to treatment necessitate special examination of this subtype (4).

Many patients undergoing gastrectomy for gastric carcinoma experience significant nutritional management challenges upon hospital discharge. The transition from structured hospital nutritional protocols to self-administered dietary care presents considerable difficulties and frequently results in compromised nutritional status. Investigational studies have demonstrated that dietary habit modification is particularly problematic when these individuals cohabitate with family members maintaining conventional nutritional regimens. Pre-operative dietary patterns likely persist into the post-operative period. Despite established evidence regarding the prognostic significance of nutritional status, these persistent behavioral patterns frequently result in patients reverting to previous suboptimal dietary regimens following surgical intervention (3).

It is well established that all cancer patients experience some degree of nutritional impairment, with this effect being particularly pronounced in patients with cancers of the upper digestive tract and pancreas, often due to mechanical impediments to food intake. The metabolic demands of cancer create a competition for nutrients between the tumor and the host, disrupting the normal functioning of the human body. Therefore, comprehensive nutritional assessment and intervention by dedicated nutritionists in oncology units are essential components of cancer care. These specialists can evaluate individual patient needs and establish appropriate nutritional treatment protocols tailored to each patient's specific requirements (5).

In this study, we aimed to investigate the effect of PNI on perioperative complications and prognosis. Additionally, we aimed to determine whether PNI varies according to tumor type by comparing it across histological subtypes.

## Materials and methods

### Data collection

Data from patients who underwent surgery for gastric cancer between 2014 and 2020 in the general surgery departments that serve as reference centers for their regions were retrospectively reviewed through patient files and electronic medical record systems.

Patients over 18 years of age, with an Eastern Cooperative Oncology Group (ECOG) performance score (6) of 0–2, histopathologically diagnosed with adenocarcinoma, who underwent curative resection, and had blood values within 1 week before surgery or before the start of neoadjuvant therapy were included in the study.

Patients with positive surgical margins, inadequate lymph node dissection, perioperative mortality, additional malignancies, irregular follow-up, missing follow-up or laboratory data, distant metastasis, ECOG performance score ≥3, incomplete neoadjuvant therapy, lack of stage-appropriate adjuvant therapy, non-adenocarcinoma cancers, HER2-positive status receiving immunomodulatory therapy, and active infection at the time of blood collection that could affect inflammatory markers were excluded from the study.

Operated patients were called for follow-up to evaluate physical examination and laboratory parameters by the oncology clinic during the first year, monthly for the first 3–6 months, and then every three months. Computed tomographic imaging covering the thorax and abdomen was performed at the 6th month and 1st year. Endoscopic evaluation was performed at the end of the first year for patients who underwent total gastrectomy, and at the sixth month and first year for those who underwent subtotal gastrectomy. After the 1st year, physical examination and laboratory analysis were performed every 6 months, while endoscopy and imaging were performed annually. In patients with histological findings suggestive of aggressive tumor behavior or surgical margin proximity, examinations were conducted at more frequent intervals on an individual basis.

Patients were examined for age, gender, body mass index (BMI), The American Society of Anesthesiologists (ASA) score (7), comorbidities, histological type of tumor (WHO classification), number of lymph nodes removed, number of positive lymph nodes, tumor location, neoadjuvant therapy status, surgical procedure (total/subtotal gastrectomy), perineural invasion (PeNI), lymphovascular invasion (LVI), follow-up duration, mortality, and laboratory parameters. According to NCCN (National Comprehensive Cancer Network) guidelines, the cancer stage is determined for gastric cancer (8). Tumor size was measured at pathological examination by opening the stomach specimens from greater or lesser curvature depending on the tumor's location. Tumor size was not measured in pathology specimens reported as linitis plastica.

Postoperative complications were systematically documented and classified according to the Clavien-Dindo scoring system, which provides a standardized approach to grading surgical complications based on the therapeutic interventions required to treat them (9). Grade I complications require no pharmacological treatment or only simple medications such as antiemetics or analgesics; Grade II complications require pharmacological intervention beyond those permitted for Grade I; Grade III complications require surgical, endoscopic, or radiological intervention (IIIa without general anesthesia, IIIb with general anesthesia); Grade IV complications are life-threatening and require Intensive Care Unit management. All complications occurring within 30 days of surgery were documented and classified according to these criteria.

When examining the response to neoadjuvant therapy, scores determined according to the modified Ryan scoring system (TRG) in pathology reports were considered (10). Patients with TRG 0-1-2 were categorized as having a response to neoadjuvant therapy, while those with TRG 3 were categorized as having no response.

Neoadjuvant therapy was administered to patients with clinical T3-T4 tumors, lymph node positivity, and locally advanced disease. The 5-Fluorouracil, leucovorin, oxaliplatin, and docetaxel (FLOT) regimen was the preferred treatment protocol. Neoadjuvant therapy was administered for a total of 4 cycles for each patient.

All laboratory data used to calculate preoperative nutritional parameters were obtained within 1 week before surgery or neoadjuvant therapy. The Prognostic Nutritional Index (PNI) was calculated using the following formula: 10 × serum albumin concentration (g/dl) + 0.005 × lymphocyte count (number/mm³) (11).

### Ethical approval

This study was conducted in accordance with the principles of the Declaration of Helsinki. Ethical approval was obtained from the Ethics Committee of the Sancaktepe Education and Research Hospital (decision number 2024/365). The requirement for informed consent was waived owing to the retrospective nature of the study.

### Statistical analysis

All statistical analyses were performed using the SPSS (Statistical Package for Social Sciences) for Windows 28.0 software. Normality was tested using the Kolmogorov–Smirnov test and graphical methods. As descriptive statistics, mean and standard deviation were used for non-parametric variables. Categorical data were expressed as counts (n) and percentages (%). The Chi-square test was employed for the comparison of two categorical variables. However, when comparing one categorical variable with a numeric value, the Mann–Whitney U test was used for the non-parametric data. Survival analyses of patients were performed using the Kaplan–Meier test. ROC analysis was performed to determine the optimal cut-off value. All statistical calculations were two-sided, and *p* < 0.05 indicated statistical significance at the confidence level of 95% (*p* < 0.05).

## Results

**Figure 1:**
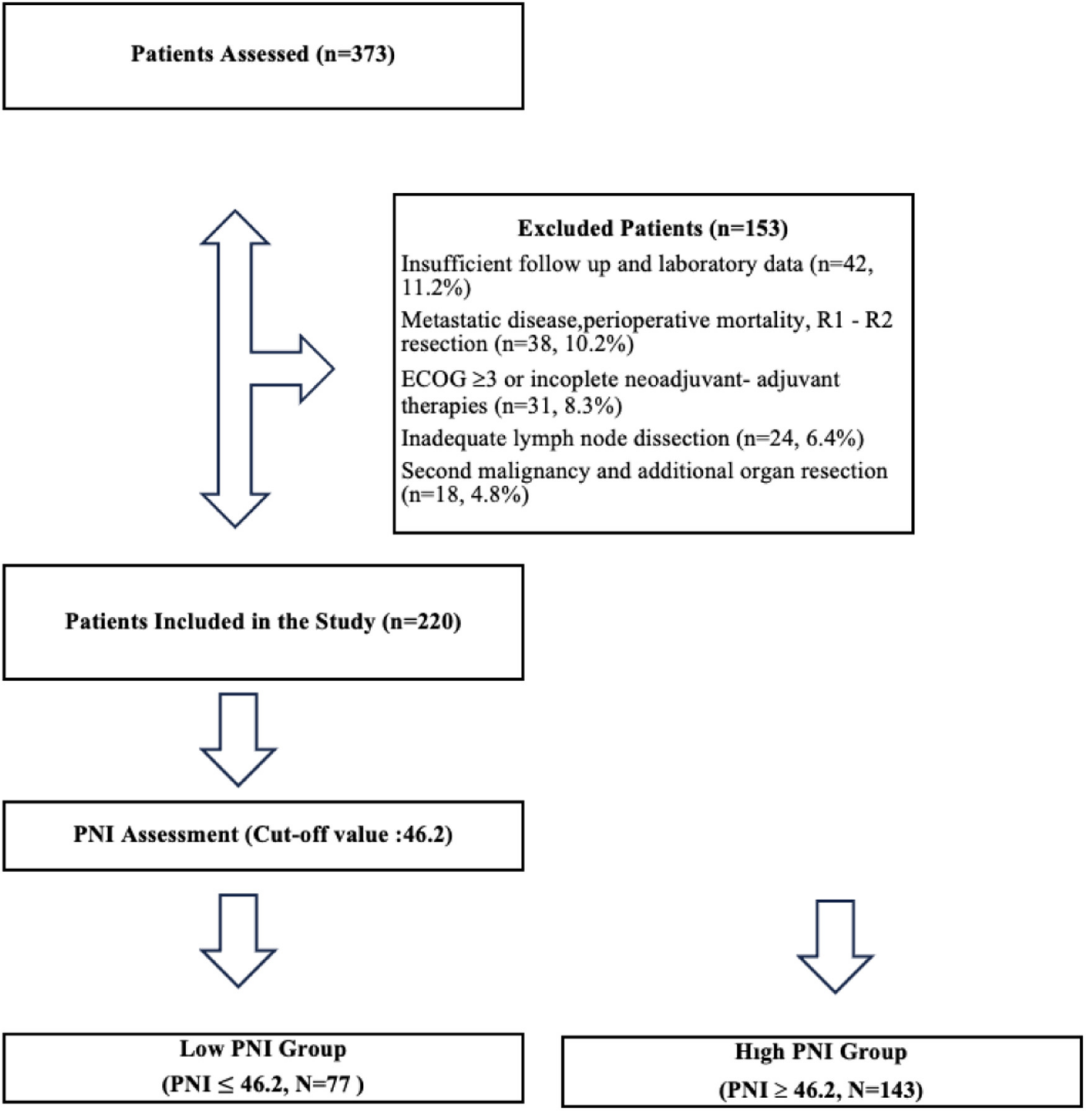
Flow-chart.

Data from a total of 373 operated patients were retrospectively screened. Forty-two patients (11.2%) were excluded due to insufficient follow-up and laboratory data, 38 patients (10.2%) due to metastatic disease detected during operation, perioperative mortality, and R1–R2 resection, 31 patients (8.3%) due to ECOG score ≥3 or failure to complete neoadjuvant-adjuvant treatments for any reason, 24 patients (6.4%) due to inadequate lymph node dissection, and 18 patients (4.8%) due to history of a second malignancy and additional organ resection. A total of 220 patients met the inclusion criteria for the study (Figure 1).

According to the normality test, age, BMI, tumor size, number of lymph nodes removed, and PNI values were not normally distributed. The mean age of all patients was 60.63 ± 10.56, BMI was 24.49 ± 3.44, and PNI value was 47.15 ± 6.07.

Of the patients, 73.2% (*n* = 161) were male and 26.8% (*n* = 59) were female; 8.2% (*n* = 18) were ASA 1, 36.8% (*n* = 81) ASA 2, 51.8% (*n* = 114) ASA 3, and 3.2% (*n* = 7) ASA 4; 9.5% (*n* = 21) were stage 1, 26.4% (*n* = 58) stage 2, and 64.1% (*n* = 141) stage 3; 29.5% (*n* = 65) had hypertension (HT), 27.3% (*n* = 60) had diabetes mellitus (DM), 6.8% (*n* = 15) had chronic obstructive pulmonary disease, and 14.1% (*n* = 31) had coronary artery disease (CAD); 20.9% (*n* = 46) had SRCC, 12.7% (*n* = 28) had well-differentiated, 30.5% (*n* = 67) had moderately differentiated, 29.5% (*n* = 65) had poorly differentiated, and 6.4% (*n* = 14) had mucinous adenocarcinoma; 57.3% (*n* = 126) had PeNI, and 63.9% (*n* = 140) had LVI. The tumor was located in the upper 1/3 in 33.6% (*n* = 74) of the patients, in the middle 1/3 in 25.9% (*n* = 57), and in the lower 1/3 in 35.9% (*n* = 79). Total gastrectomy was performed in 64.1% (*n* = 141) and subtotal gastrectomy in 35.9% (*n* = 79) (Table 1).

**Table 1:** Demographic and clinicopathologic distribution of patients.

Variables		
Stage, *n*, %	Stage I	21 9.5%
	Stage II	58 26.4%
	Stage III	141 64.1%
Gender	Male	161 73.2%
	Female	59 26.8%
ASA score	ASA I	18 8.2%
	ASA II	81 36.8%
	ASA III	114 51.8%
	ASA IV	7 3.2%
HT	Yes	65 29.5%
	No	155 70.5%
DM	Yes	60 27.3%
	No	160 72.7%
COPD	Yes	15 6.8%
	No	205 93.2%
CAD	Yes	31 14.1%
	No	189 85.9%
Localization	Upper 1/3	74 33.6%
	Middle 1/3	57 25.9%
	Lower 1/3	79 35.9%
	Linitis plastica	10 4.5%
Grade	Well	28 12.7%
	Moderate	67 30.5%
	Poor	65 29.5%
	Signet cell	46 20.9%
	Mucinous	14 6.4%
NAC	Yes	128 58.2%
	No	92 41.8%
5 year survival	Yes	154 70.0%
	No	66 30.0%
LVI^a^	Yes	140 63.9%
	No	79 36.1%
PeNI	Yes	126 57.3%
	No	94 42.7%
Surgery	Total	141 64.1%
	Subtotal	79 35.9%
Clavien-Dindo complications	No complication	129 58.6%
	Grade I	43 19.5%
	Grade II	25 11.4%
	Grade III	17 7.7%
	Grade IV	6 2.7%
Age	Mean ± sd	60.3 ± 10.56
BMI (kg/m^2^)	Mean ± sd	24.49 ± 3.44
Lymph node	Mean ± sd	25.0 ± 8.04
Tumor size (cm)	Mean ± sd	5.40 ± 1.99
PNI	Mean ± sd	47.15 ± 6.07

^a^ Missing case ASA, American Society of Anesthesiology; HT, hypertension; DM, diabetes; COPD, chronic obstructive pulmonary disease; CAD, coronary artery disease; NAC, neoadjuvant chemotherapy; LVI, lymphovascular invasion, PeNI, perineural invasion; sd, standard deviation.

**Figure 2:**
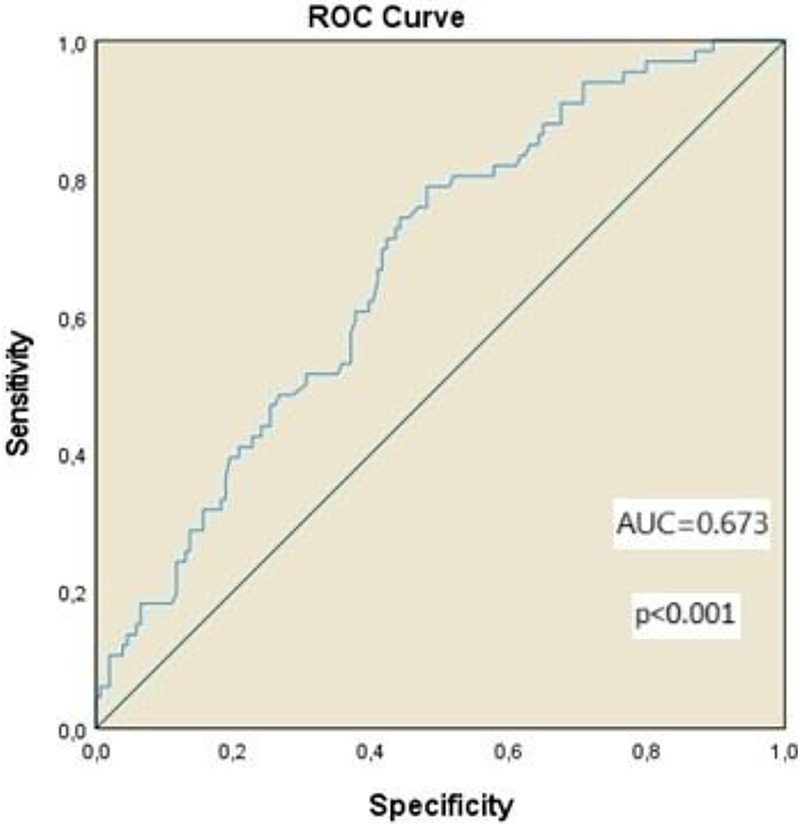
ROC curve of PNI for 5-year OS. (AUC = 0.673, 95%CI: 0.599–0.747, *p* < 0.001; sensitivity 78.8%, specificity 51.9%).

In the ROC analysis performed to find the cut-off value for evaluating 5-year overall survival (OS) for PNI, the PNI value was found to be 46.2 (AUC = 0.673, 95%CI: 0.599–0.747, *p* < 0.001; sensitivity 78.8%, specificity 51.9%) (Figure 2).

**Figure 3:**
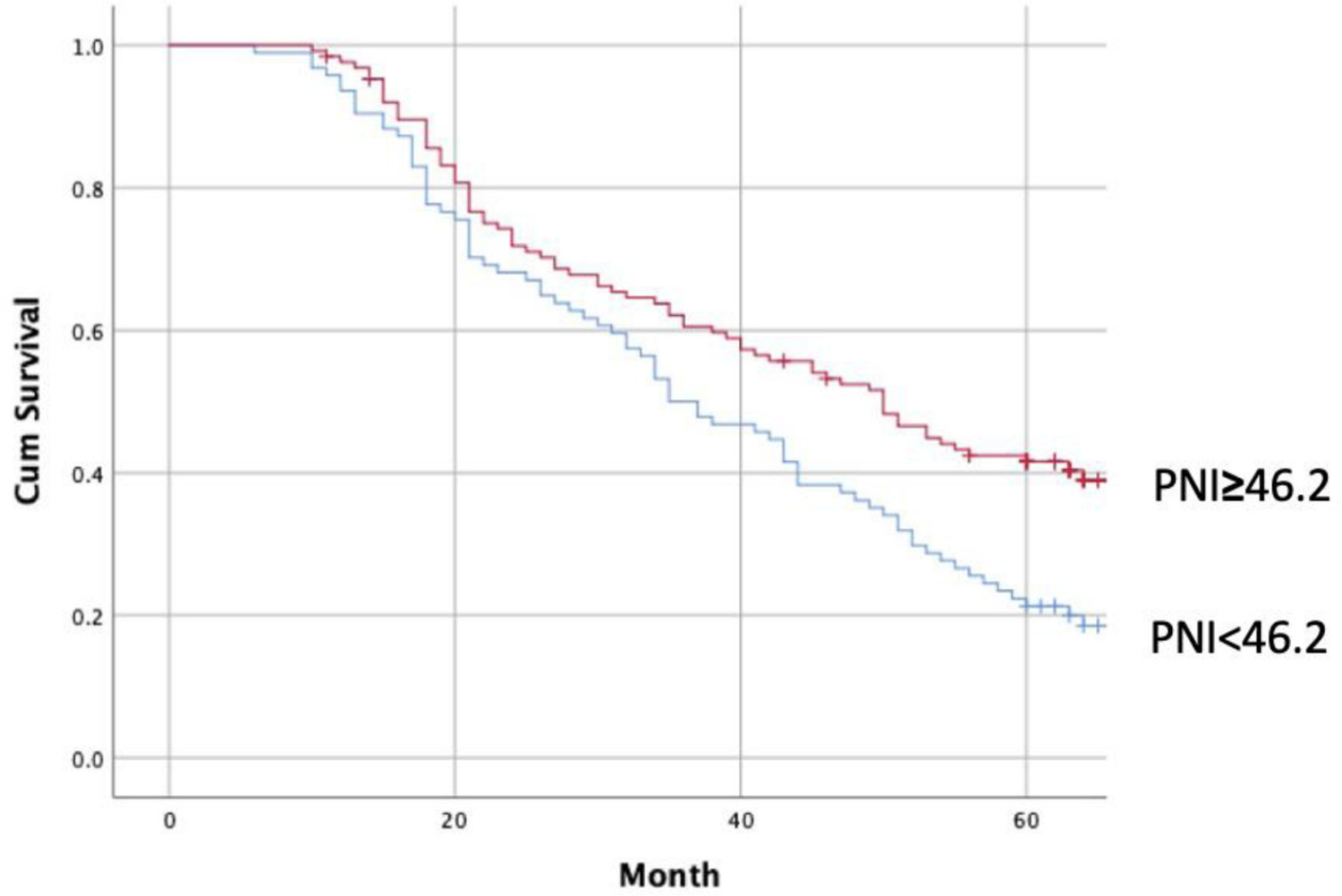
Survival analysis between Low and high PNI groups. (Log Rank p:0.001).

The 5-year survival rate was found to be 30.0%. When comparing the survival of patients with PNI values less than 46.2 and those greater than 46.2, it was observed that survival was worse in patients with low nutritional index (log rank *p* = 0.001) (Figure 3).

In the examination conducted by dividing patients with PNI values less than 46.2 as poor and those with values greater than 46.2 as good nutritional status into two categories, it was observed that PNI was lower in the group where PeNI and LVI were more common (p values 0.005 and 0.032, respectively). Patients with SRCC had statistically lower PNI compared to other adenocarcinoma types (*p* = 0.001). Between the two groups, 5-year survival was statistically worse in the low PNI group (*p* < 0.001). There was no statistically significant difference between response to neoadjuvant therapy and PNI value in patients receiving neoadjuvant therapy (*p* = 0.095). When comparing complications according to the Clavien-Dindo classification, Grade 1 (23.4% vs. 16.7%), Grade 2 (16.0% vs. 7.9%), Grade 3 (11.7% vs. 4.8%), and Grade 4 complication rates were higher in the low PNI group, whereas patients without complications were higher among the high PNI group (44.7% vs. 69.0%) (*p* = 0.006) (Table 2).

**Table 2:** Evaluation of patients Due to Cut-off value of PNI.

Variables	Category	PNI				*p*
		<46.2		≥46.2		
Stage	Stage I	6	6.4%	15	11.9%	0.379
	Stage II	25	26.6%	33	26.2%	
	Stage III	63	67.0%	78	61.9%	
PeNI	No	22	28.6%	72	50.3%	0.005^a^
	Yes	55	71.4%	71	49.7%	
LVI	No	20	26.3%	59	41.3%	0.032^a^
	Yes	56	73.7%	84	58.7%	
NAC response	Regression	19	42.2%	49	59.0%	0.095
	Progression	26	57.8%	34	41.0%	
Histology	RGC	54	70.1%	119	83.2%	0.001^a^
	SCGC	23	29.9%	24	16.8%	
5 year OS	No	90	62.9%	58	75.3%	<0.001^a^
	Yes	53	37.1%	19	24.7%	
Clavien-Dindo	No complicaton	42	44.7%	87	69.0%	0.006^a^
	Grade I	22	23.4%	21	16.7%	
	Grade II	15	16.0%	10	7.9%	
	Grade III	11	11.7%	6	4.8%	
	Grade IV	4	4.3%	2	1.6%	

PNI, prognostic nutritional index; SII, systemic immun-inflamatuar index; PeNI, perineural invasion; LVI, lymphovascular invasion; NAC, neoadjuvant chemotheraphy; SCGC, signet cell gastric adenocarcinomas; RGC, remaining gastric adenocarcinomas; OS, overall survival.

^a^ Statistical significance at the 95% confidence level.

In the logistic regression analysis based on 5-year survival, while chronic obstructive pulmonary disease (COPD), tumor stage, LVI, PeNI, histological type being SRCC, and low PNI values were significant according to univariate analysis, in multivariate analysis, tumor stage (for stage I *p* = 0.01, for stage 2 *p* = 0.034, and for stage 3 *p* = 0.002) and PNI value (*p* = 0.001) stood out as the only parameters affecting 5-year survival (Table 3).

**Table 3:** Logistic regression analysis for 5-year overall survival.

Variables		OR	95% C.I Lower	95% C.I Upper	P (univariate)	P (multivariate)
Gender		0.925	0.480	1.783	0.816	–
HT		0.775	0.416	1.442	0.421	–
DM		1.360	0.693	2.667	0.371	–
COPD		0.257	0.087	0.754	**0**.**013**^a^	0.076
CAD		0.633	0.288	1.394	0.213	–
Stage	Stage I	–	–	–	**<0**.**001**^a^	**0**.**001**^a^
	Stage II	0.467	0.164	1.325	**0**.**015**^a^	**0**.**034**^a^
	Stage III	0.103	0.037	0.281	**<0**.**001**^a^	**0**.**002**^a^
ASA Score	ASA 1	1.500	0.375	5.998	0.566	–
	ASA 2	1.143	0.291	4.491	0.848	
	ASA 3	1.714	0.285	10.303	0.556	
	ASA 4	–	–	–	0.777	
Localization	Upper 1/3	1.436	0.664	3.103	0.358	–
	Middle 1/3	1.804	0.895	3.637	0.101	
	Lower 1/3	0.346	0.041	2.918	0.329	
	LP	–	–	–	–	
NAC		0.569	0.318	1.018	0.057	–
LVI		0.312	0.171	0.570	**<0**.**001**^a^	0.891
PeNI		0.421	0.234	0.758	**0**.**004**^a^	0.454
HistologicL Type		0.338	0.143	0.801	**0**.**014**^a^	0.560
Age		0.998	0.971	1.026	0.896	–
BMI		1.086	0.998	1.183	0.146	–
Dissected Lypmph Node		0.998	0.963	1.035	0.912	–
Tumor Size (cm)		0.927	0.798	1.077	0.324	–
PNI		1.118	1.060	1.179	**<0**.**001**^a^	**0**.**001**^a^

^a^ Statistical significance at the 95% confidence level; BMI, body-mass index; PNI, prognostic nutritional index; ASA, American Society of Anesthesiology; HT, hypertension; DM, diabetes; COPD, chronic obstructive pulmonary disease; CAD, coronary artery disease; NAC, neoadjuvant chemotheraphy; LVI, lymphovascular invasion; PeNI, perineural invasion; LP, linitis plastica.

## Discussion

Gastric cancer is a complex clinical condition requiring the development of individualized strategies in treatment approaches, especially considering the factor of malnutrition. This study demonstrates not only the value of the Prognostic Nutritional Index (PNI) in predicting postoperative complications and long-term survival in patients with gastric cancer but also highlights the differences among histological subtypes. Particularly, the association of signet ring cell carcinoma (SRCC) with lower PNI values compared to other adenocarcinoma subtypes emphasizes the necessity of closer monitoring of nutritional status in the treatment of this aggressive subtype. The results of the study indicate that preoperative PNI assessment could be a valuable parameter in predicting patients’ risk of postoperative complications and forecasting long-term survival, thus helping clinicians in planning patient-centered treatment approaches.

Nutritional status inevitably affects tumor patients’ prognosis through their resistance to the tumor's catabolic activity. An increasing number of studies show that patients’ basic nutritional status is associated with long-term prognosis (12). Inadequate nutrition, decreased immunity, and increased inflammation not only affect cancer patients’ response to treatment but may also increase the likelihood of recurrence and metastasis of malignant tumors (13). Based on this, the Prognostic Nutritional Index (PNI), which reflects both the immune status (lymphocytes) and nutritional reserves (albumin) of patients, provides information about the overall physiological condition of patients. This simplicity confers a significant advantage in terms of ease of use (14).

PNI was first reported by Onodera et al. in 1984 to have prognostic value in patients undergoing surgery for gastrointestinal cancer (15). Since then, numerous studies have been conducted on this topic. It was reported that preoperative PNI was a good prognostic indicator of hepatocellular carcinoma, gastric cancer, colorectal carcinoma, and pancreatic cancer (16). A 2024 meta-analysis examining 18,596 patients found a significant relationship between PNI and OS (17). In our study, consistent with the literature, low PNI was associated with poor prognosis, and in multivariate analysis, it was found to be a risk factor affecting survival on its own.

There are various studies investigating the cut-off value for PNI, but there is no consensus on this issue. Some studies accept 45 as the cut-off value, as stated in Onodera's study (18, 19). However, when the literature is examined, <43, <44.8, ≤49.2, <52, and <52.9 have been found to be associated with poor prognosis (17). In our study, similar to the value in the original study, <46.2 with sensitivity 78.8% and specificity 51.9% was found to be associated with poor prognosis.

An inverse relationship is observed between PNI and the pathological stage of the tumor (20). According to a meta-analysis conducted in 2016, it was stated that there is a correlation between low PNI values and advanced pathological stage. However, according to the same meta-analysis, a direct relationship between stage and PNI could not be demonstrated in 5 different studies examined. In our study, while a direct relationship was found between stage and OS, no statistically significant relationship was found between stage and PNI (19).

In addition, it has been shown that there is a relationship between LVI and PeNI and PNI; PeNI and LVI were more common in patients with low PNI (19, 21). In a similar study published in 2024, no statistically significant relationship was found between PeNI and LVI in patients with PNI > 39.8 (11). In our study, PeNI and LVI were found to be more common in patients with lower PNI. Additionally, in our study, PNI was found to be lower in patients diagnosed with gastric cancer with signet ring cell histology compared to the other group. In light of the studies examined, it is understood that there is a relationship between these parameters, which are related to tumor aggressiveness and have been proven by various studies to have a negative effect on survival, and PNI. Probably, PNI values are found to be lower because the catabolic process is more intense in tumors with more aggressive behavior (22, 23). Prospective studies with large patient populations are needed to resolve conflicting findings on this issue.

There are studies examining the relationship between pre-neoadjuvant nutritional status and response to neoadjuvant therapy in gastric cancer. In a study published in 2024, it was found that patients with low PNI values had worse responses to neoadjuvant therapy (24). In their article published in 2021, Meng F. et al. developed a scoring system combined with a systemic immune-inflammatory index in addition to PNI, and according to this, they state that patients with low PNI values respond worse to neoadjuvant therapy (25). In another study, the frequency of pathological complete response was found to be higher in non-metastatic gastric cancer patients with better immune-nutritional status, but no statistical difference was found (6.6% vs. 1.2%, *P* = 0.107) (26). In our study, regression was observed in 59.2% of those receiving neoadjuvant therapy in the high PNI group and in 44.2% of patients in the low PNI group, but the effect of PNI value calculated before neoadjuvant therapy on the response to neoadjuvant therapy was not statistically determined (*p* = 0.095). It is seen that nutritional status is related to the response to neoadjuvant therapy, but additional studies are needed to determine whether PNI values can be used to predict response to neoadjuvant therapy.

PNI value also has an effect on surgical complications in the postoperative period. In our study, complication rates according to the Clavien-Dindo classification were found to be significantly higher in the patient group with PNI < 46.2 (*p* = 0.006). In a meta-analysis published by Yang et al. in 2016, including 3396 gastric cancer patients, a strong association was demonstrated between low PNI values and postoperative complications (OR = 1.74, 95% CI = 1.41–2.16, *p* < 0.01). In this meta-analysis, pooled results from five different studies revealed that patients with poor nutritional status were more vulnerable to postoperative complications (19). In the current literature, no study reporting the absence of a relationship between PNI and postoperative complications has been found. Similarly, in our study, while Grade 1 (23.4% vs. 16.7%), Grade 2 (16.0% vs. 7.9%), Grade 3 (11.7% vs. 4.8%), and Grade 4 complication rates were higher in the low PNI group, the rate of patients without complications was higher in the high PNI group (44.7% vs. 69.0%). This suggests that well-nourished patients may be better able to tolerate the immunosuppression associated with circulating inflammatory cytokines that could be induced by post-operative complications (27). Therefore, when planning surgical treatment for gastric cancer patients, preoperative assessment of PNI value can be used as a valuable parameter in predicting the risk of postoperative complications and planning patient-specific approaches.

In our study, the PNI value in patients receiving neoadjuvant therapy is the PNI value calculated from blood taken before the start of neoadjuvant therapy. It may be thought that taking the PNI value before treatment in the group receiving neoadjuvant therapy and before operation in the group not receiving neoadjuvant therapy may harm homogeneity, but in patients who underwent surgery after neoadjuvant therapy, there was a relationship between the PNI value calculated before neoadjuvant chemotherapy and OS, while no relationship was found between the PNI value calculated in blood taken before surgery after neoadjuvant therapy and OS. The probable reason for this is the hematological toxicity of chemotherapeutic agents and bone marrow suppression. Therefore, it has been stated that it would be more appropriate to use the value taken before neoadjuvant therapy in order to use PNI as a prognostic indicator (28).

The clinical implications of our findings extend beyond mere prognostic assessment. Given that lower PNI values correlate with increased complications and reduced survival, the role of nutritional intervention becomes paramount. In the pre-neoadjuvant or preoperative phase, ensuring optimal nutritional status through daily nutritionist consultation and intervention is crucial. The availability of specialized immunonutrition formulations provides additional tools for optimizing patient outcomes. Our results reinforce the principle that improved nutritional status directly translates to better treatment outcomes, emphasizing the need for proactive nutritional management as an integral component of gastric cancer treatment protocols.

Future prospective studies should consider incorporating serial PNI measurements during the postoperative period to evaluate the dynamic changes in nutritional status and their impact on long-term outcomes. Such longitudinal assessment could provide valuable insights into the optimal timing and effectiveness of nutritional interventions.”

There are some limitations in our study. Primarily, our study is a retrospective study. Recurrence timing and disease-free survival analysis could not be performed because all data on patients’ adjuvant treatment protocols and durations and recurrence timing could not be fully accessed. However, in the survival analysis performed, only tumor-related deaths were referenced. The sensitivity and specificity of the detected cut-off value are not sufficient, but there is no study in the literature that finds a cut-off value with high sensitivity and specificity.

Due to sample size limitations and the retrospective nature of the study, propensity score matching analysis could not be performed to create matched cohorts based on PNI and tumor stage.

Despite these limitations, our study provides valuable information on 5-year OS, surgical complications, and nutritional status according to histological subtypes with PNI and contributes to the literature.

## Conclusion

In conclusion, PNI is one of the parameters that can determine survival in gastric cancer. Major surgical complications are more common in patients with low PNI. It is observed that low PNI values are associated with SRCC, LVI, and PeNI, which have a worse prognosis. It is observed that PNI decreases directly proportional to tumor aggressiveness. However, it is evident that prospective studies with large patient populations are needed both to increase the reliability of this parameter and to find an optimal cut-off value. We believe that conducting prospective studies with large populations regarding parameters such as PNI and including these parameters in the evaluation is important for planning patient-specific treatment.

## Data Availability

The raw data supporting the conclusions of this article will be made available by the authors, without undue reservation.
